# The repeatability of bilateral diffusion tensor imaging (DTI) in the upper leg muscles of healthy adults

**DOI:** 10.1007/s00330-019-06403-5

**Published:** 2019-11-08

**Authors:** Jithsa R. Monte, Melissa T. Hooijmans, Martijn Froeling, Jos Oudeman, Johannes L. Tol, Mario Maas, Gustav J. Strijkers, Aart J. Nederveen

**Affiliations:** 1 Amsterdam UMC, Radiology & Nuclear Medicine, University of Amsterdam, Meibergdreef 9, Amsterdam, The Netherlands; 2 Amsterdam UMC, Biomedical Engineering and Physics, University of Amsterdam, Meibergdreef 9, Amsterdam, The Netherlands; 3 Department of Radiology, University Medical Center Utrecht, Utrecht, The Netherlands; 4 Amsterdam UMC, Department of Orthopaedic Surgery, University of Amsterdam, Meibergdreef 9, Amsterdam, The Netherlands

**Keywords:** Diffusion tensor imaging, Magnetic resonance imaging, Hamstring muscles, Quadriceps muscle

## Abstract

**Objectives:**

Assessment of the repeatability of diffusion parameter estimations in the upper leg muscles of healthy adults over the time course of 2 weeks, from a simultaneous bilateral upper leg DTI measurement.

**Methods:**

SE-EPI DTI datasets were acquired at 3 T in the upper legs of 15 active adults at a time interval of 2 weeks. ROIs were manually drawn for four quadriceps and three hamstring muscles of both legs. The following DTI parameters were analyzed: 1st, 2nd, and 3rd eigenvalue (*λ*_1_, *λ*_2_, and λ_3_), mean diffusivity (MD), and fractional anisotropy (FA). DTI parameters per muscle were calculated with and without intravoxel incoherent motion (IVIM) correction together with SNR levels per muscle. Bland-Altman plots and within-subject coefficient of variation (wsCV) were calculated. Left-right differences between muscles were assessed.

**Results:**

The Bland-Altman analysis showed good repeatability of all DTI parameters except FA for both the IVIM-corrected and standard data. wsCV values show that MD has the highest repeatability (4.5% IVIM; 5.6% standard), followed by *λ*_2_ (4.9% IVIM; 5.5% standard), *λ*_1_ (5.3% IVIM; 7.5% standard), and *λ*_3_ (5.7% IVIM; 5.7% standard). wsCV values of FA were 15.2% for the IVIM-corrected data and 13.9% for the standard analysis. The SNR (41.8 ± 16.0 right leg, 41.7 ± 17.1 left leg) and wsCV values were similar for the left and right leg and no left-right bias was detected.

**Conclusions:**

Repeatability was good for standard DTI data and slightly better for IVIM-corrected DTI data. Our protocol is suitable for DTI of the upper legs with overall good SNR.

**Key Points:**

*• The presented DTI protocol is repeatable and therefore suitable for bilateral DT imaging of the upper legs.*

*• Additional B1* ^*+*^ *calibrations improve SNR and repeatability.*

*• Correcting for perfusion effects improves repeatability.*

**Electronic supplementary material:**

The online version of this article (10.1007/s00330-019-06403-5) contains supplementary material, which is available to authorized users.

## Introduction

Muscle injuries are the most prevalent injuries in elite and recreational sports [1]. Although considerable progress has been made, effective treatment and rehabilitation of muscle injury, particularly those affecting leg muscles, remain challenging as demonstrated by the high recurrence incidence after acute hamstring injury [2, 3]. For diagnostic purposes, conventional T2-weighted fat-suppressed MRI is commonly used [4]. Unfortunately, a major drawback is that hyperintensity—a characteristic of the injury—remains visible in these images long after all clinical symptoms have cleared and the athlete has resumed sports activity [5]. This implies that T2-weighted imaging with fat suppression is insufficient for evaluating muscle recovery during rehabilitation and for assessing return to play (RTP) [6–8]. More sensitive and quantitative methods are therefore highly desired.

Over the past decades, diffusion tensor imaging (DTI) MRI emerged as a promising technique to evaluate muscle tissue repair [9]. Previous work showed that DTI facilitated detection of structural changes in muscle beyond conventional T2-weighted MRI [10]. Also, research performed in injured athletes showed that DTI has the potential for the assessment of muscle injuries [11, 12].

Despite its promise for diagnosis and follow-up of sports-related muscle injury, repeatability of the muscle DTI method needs to be established and a couple of experimental hurdles need to be overcome. First, most DTI studies with 3-T MRI systems targeting the upper legs have been performed in a single left or right leg, because imaging of both legs resulted in varying image quality stemming from B1 inhomogeneities [13]. However, a bilateral assessment is important as the contralateral leg serves as an internal reference to control for between-subject variations in DTI indices introduced by differences in age, gender, and activity level [14, 15]. Bilateral imaging therefore will significantly increase the discriminative power of the technique, provided DTI of both legs simultaneously provides sufficient image quality to derive accurate and repeatable quantitative diffusion indices [16, 17]. Secondly, signal attenuation originating from blood flow in the microvascular network (perfusion) can be a confounding factor [18, 19]. Previous research has shown that modeling the signal attenuation with a so-called intravoxel incoherent motion (IVIM) model improves DTI parameter estimations and potential repeatability [20].

The combined main aims of this study were therefore to assess the repeatability of diffusion parameter estimations in the upper leg muscles of healthy active from a simultaneous bilateral upper leg DTI measurement, with and without IVIM correction. Furthermore, we also considered the benefits of additional B1^+^ calibrations. We hypothesized that additional prescans prior to DTI could improve B1^+^ homogeneity [21].

## Methods and materials

This study was waived by the local IRB as no patients participated. We obtained written informed consent from all volunteers prior to the study.

### Subjects

Eighteen healthy subjects, 16 males and 2 females, mean age 28.4 ± 5.2 years (range 22–40 years) with no recorded upper leg injury in the half-year prior to this study were recruited. Inclusion criteria were the following: healthy, at least 18 years old, and active in sports. Subjects engaged in sports activities on average 2.9 times per week and were instructed to continue with their sport/exercise regimen. Exclusion criteria were the following: MRI contra-indications, injury of one of the leg muscles seen on MRI, or unable to fully complete both scanning sessions. The protocol was performed twice with a 2-week interval (average 14 days, range 13–15 days). One subject was excluded based on a muscle injury detected at the first scanning session and two subjects had incomplete datasets due to scheduling conflicts. Thus, 15 subjects (13 males, 2 females) were finally included.

### MRI

MRI was acquired with a 3-T Philips Ingenia MRI scanner (Philips Healthcare) using a 16-channel receive array coil placed on top of the legs in combination with 12 receive coils in the patient table. Subjects were placed supine and feet first in the scanner. Dielectric pads were placed on top of the left upper leg and under the right upper leg [13]. These pads served to reduce RF inhomogeneity by augmenting the applied B1^+^ field. The geometrical planning for the second session was carefully matched to one of the first session using anatomical landmarks and saved images of the slice planning.

The MRI examination consisted of four sequences:

1. Axial DTI sequence with multiple *b* values for the purpose of IVIM correction [20];
2. Axial T2-weighted sequence for anatomical reference;
3. Axial PD-weighted DIXON sequence for the purpose of segmentation;
4. Coronal T2-weighted sequence to assess potential muscle injury (exclusion criterion).

Total scanning time was 25 min. Table 1 lists detailed scanning parameters. Additional DTI parameters: sequence = diffusion-weighted spin-echo - echo-planar-imaging (SE-EPI), fat suppression = SPAIR (spectral attenuated inversion recovery), no slice gap, fold-over direction = anterior-posterior, *b* values (no. gradient orientations) = 0 (1×), 1 (8×), 5 (3×), 10 (3×), 20 (3×), 50 (3×), 100 (3×), 200 (10×), 400 (10×), 600 (12×) s/mm^2^ (10 b values, 56 gradient directions), scan duration 11 m 8 s, receiver bandwidth 27.9 Hz/pixel, echo times of the axial PD-weighted DIXON sequence: 1.35 ms, 2.45 ms, 3.55 ms, 4.65 ms, 5.75 ms, and 6.85 ms.

**Table 1:** MRI scan parameters

Sequence	OR	FOV (mm^2^)	VOX (mm^3^)	TR/TE (ms)	NSA	Echo	Slices	PIF
DTI (SE-EPI)	AX	480 × 252	3 × 3 × 5	5914/54.56	2	1	40	1.5
T2 (MS_TSE)	AX	480 × 252	1.5 × 1.5 × 2.5	2000/70	1	1	80	2.8
PD DIXON (FFE)	AX	480 × 252	1.5 × 1.5 × 2.5	500/1.35	3	6	80	3
T2 (MS_TSE)	COR	450 × 450	0.55 × 0.66 × 4	2000/60	1	1	90	2.8

*AX*, axial; *COR*, coronal; *FOV*, field of view; *VOX*, voxel dimensions; *NSA*, number of signal averages; *TR*, repetition time; *TE*, echo time; *PIF*, parallel imaging factor; *OR*, orientation

### IVIM correction

For the IVIM-corrected data, first, the full IVIM model was fitted to the average signal per *b* value using NLS model (initialization parameters, S0 = 1; *f* = 0.05; *D* = 0.003; *D*p = 0.1). The isotropic perfusion component of the signal is calculated and subtracted from the signal of all diffusion directions and *b* values. The perfusion-corrected signal of all *b* values and directions was then used for tensor estimation using an iterative weighted linear least squares (iWLLS) algorithm [20].

### B1^+^ calibration

The 30-s prescans included a B1^+^ calibration scan, a SENSE reference scan, and a coil survey scan. The prescans are proprietary Philips technology and are currently performed automatically on Philips Ingenia Systems (since release R5.3.0) at the beginning of the protocol and any time when a substantial change in the field of view or movement of the scanner table occurs. We performed additional prescans prior to the DTI sequence for 5 subjects.

### Analysis

DTI data were processed using the custom-built software DTITools (https://github.com/mfroeling/DTITools) for Wolfram Mathematica 11.3. First, the diffusion-weighted images were pre-processed using a principle component analysis (PCA) noise suppression algorithm [22]. Subsequently, the diffusion-weighted images were registered to the T2-weighted axial images, using rigid and b-spline transformations [23] to correct for subject motion and image deformations due to eddy currents. An iterative weighted linear least squares algorithm [24] was used for the estimation of *λ*_1_, *λ*_2_, and *λ*_3_ from which mean diffusivity (MD) and fractional anisotropy (FA) were calculated. Diffusion attenuation was either modeled using a single exponential decay model—this will be referred to as “standard”—or by IVIM modeling to correct the diffusion parameters for the effects of pseudo-diffusion (perfusion) [20]. The latter will be referred to as “IVIM-corrected.” Signal-to-noise ratio (SNR) per muscle was estimated to assess DTI quality. The standard deviation (*σ*) of the noise was mapped through the PCA denoising process [22]. This *σ* map was used for the SNR estimations [25]. SNR was defined as the mean of the signal in a muscle ROI divided by *σ*. In case of SNR < 20, this muscle was excluded from further analysis [16].

**Fig. 1:**
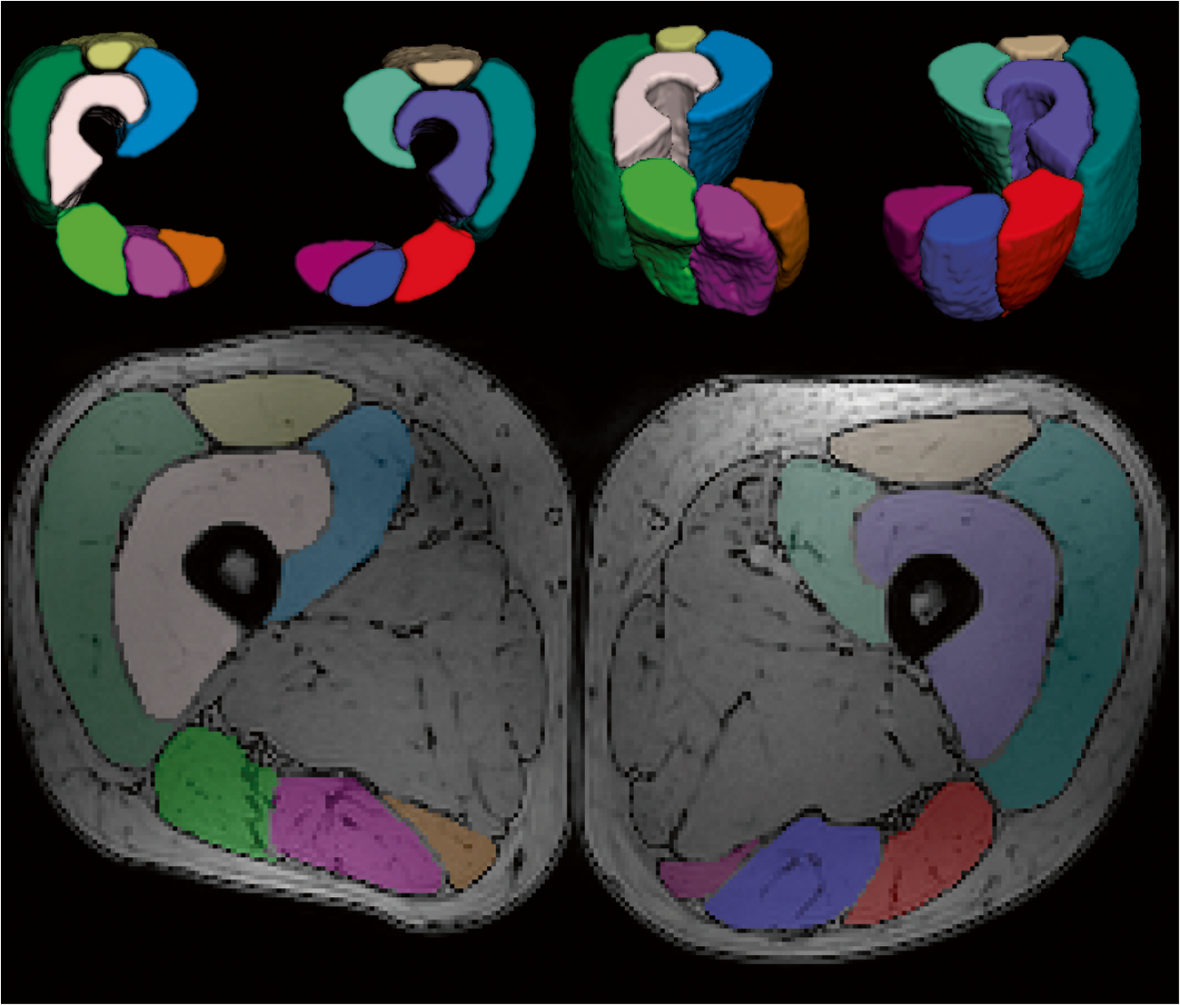
**a** Representative axial proton density–weighted MRI with overlaid muscle segmentations in color. **b** 3D volume rendering of segmented muscles in the center 20 slices of the upper leg

The analysis was done for the following muscles of the right (R) and left (L) legs: rectus femoris (RF), vastus medialis (VM), vastus intermedius (VI), vastus lateralis (VL), semitendinosus (ST), biceps femoris long head (BF), and semimembranosus (SM). Manual segmentation of the muscles was performed in ITK-snap [26] (www.itksnap.org) in the out-of-phase PD-weighted DIXON (axial) images. The segmentation was performed slice by slice, carefully excluding subcutaneous fat (Fig. 1). The segmentations were imported in Mathematica as masks, eroded 1 pixel, and rescaled to the resolution of the DTI images for the quantification of the DTI parameters and SNR per muscle. The mean value of the estimated DTI indices was calculated over the 20 middle slices. The injury in our clinical study is always placed in the middle of the field of view planning. Hence, we are interested in the quality and repeatability of the 20 middle slices of the sequence.

### Statistical analysis

The repeatability of the two measurements was assessed with Bland-Altman plots and calculations of the within-subject coefficient of variation (wsCV), which was calculated as the ratio of the standard deviation of the paired differences to the mean.

The statistical analysis was performed for the standard and IVIM-corrected data, and separately for the right and left legs. Paired samples *t* tests were done to compare the wsCV values per DTI parameter for the IVIM-corrected data with standard data. The same approach was used to compare wsCV values per DTI parameter between the subgroups of 5 and 10 subjects for the IVIM-corrected data. Significance was set at *p* < 0.05. The minimal detectable difference, defined as 1.96 × standard deviation of the paired differences, was calculated per muscle per DTI parameter.

## Results

### Data quality

SNR calculations for 420 muscles (15 participants × 7 muscles per leg × 2 legs × 2 time points) resulted in the exclusion of 50 (11.9%) muscles due to insufficient SNR (< 20) leaving 185 muscle pairs (370) for further analysis. Mean SNR for all included muscles was 44.5 ± 8.9.

Mean SNR of all muscles was 41.8 ± 16.0 for the right and 41.7 ± 17.1 for the left leg. An overview of the numbers of included and excluded muscles per subject, as well as mean SNR values, is given in Supplemental Tables 1 and 2.

The visual assessment showed that, despite fat suppression and B1^+^ shimming, chemical shift artifacts and shading artifacts (B1^+^-field inhomogeneities) were present in a majority of the images (Supplemental Figure 1). The artifacts were mostly present in the RF muscle, causing an overall SNR drop in the quadriceps muscles compared with that in the hamstring muscles. The shading artifacts were primarily present in the upper quadrant of the left leg (quadriceps) and in the lower quadrant of the right leg (hamstrings) and were most apparent in the left quadriceps. These artifacts caused SNR differences between left and right with higher SNR in the left hamstrings and quadriceps (Supplemental Table 2).

**Fig. 2:**
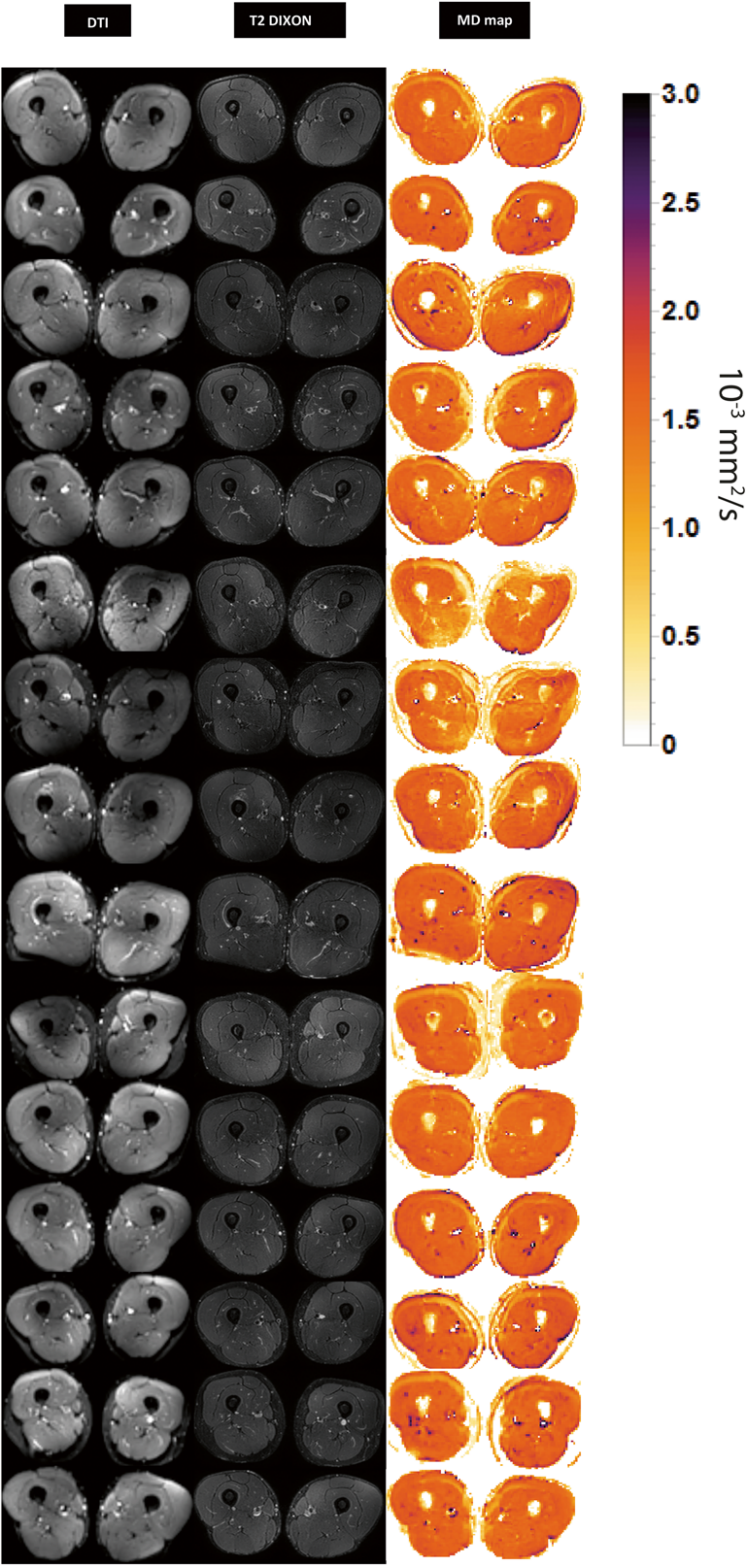
Representative axial slice of the DTI (*b* value 0 s/mm^2^) images, T2-weighted images, and MD maps for all 15 subjects at the first time point

**Fig. 3:**
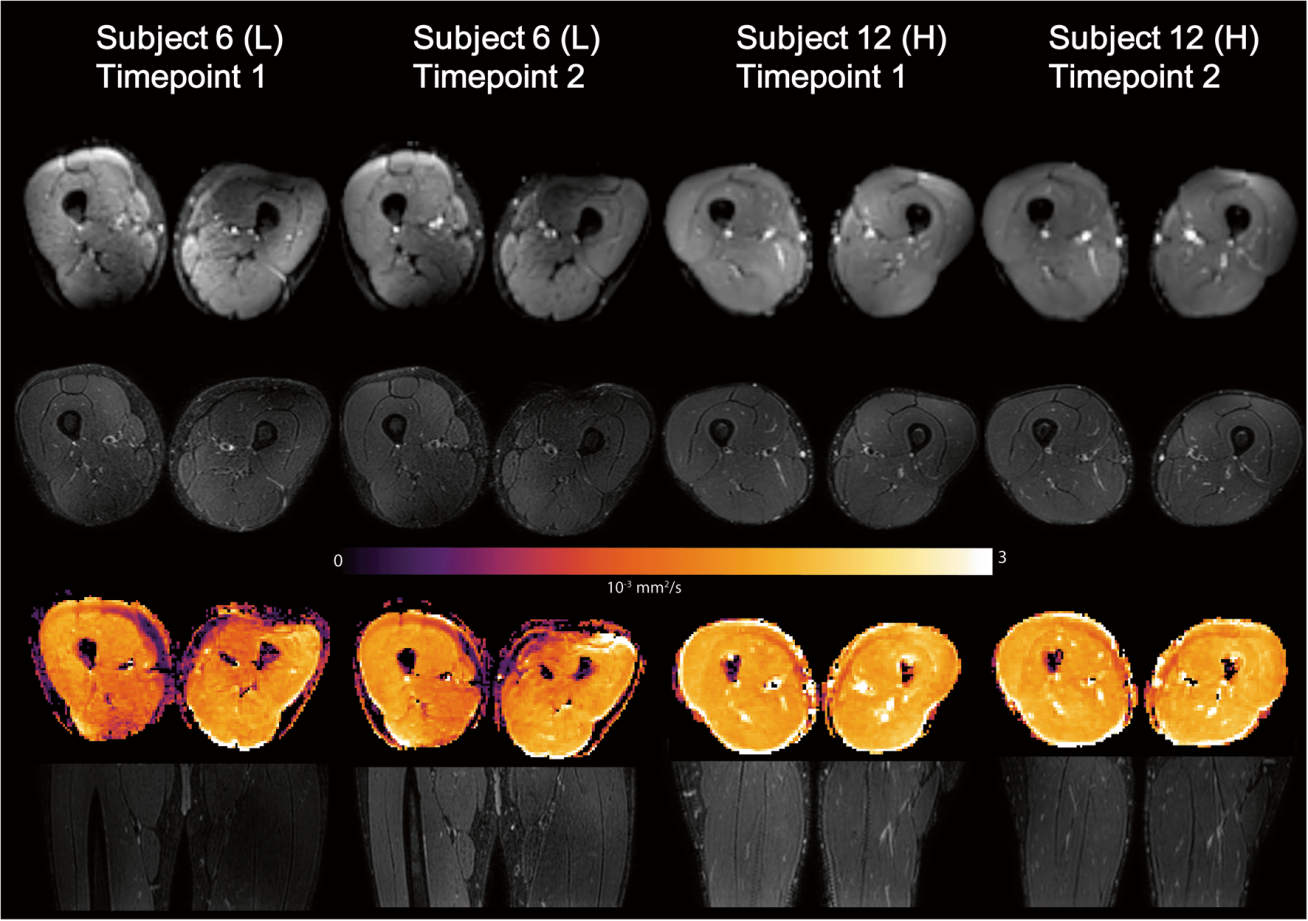
First and second time point scans of two subjects with (L) the lowest SNR and (H) typical high SNR. From top to bottom: axial DTI (*b* value = 0 s/mm^2^), T2-weighted images, MD map, coronal T2-weighted images. Mean SNR subject 6: time point 1, 24.8 ± 14.4; time point 2, 27.9 ± 13.4. Mean SNR subject 12: time point 1, 47.6 ± 11.5; time point 2, 54.2 ± 14

Despite these regional artifacts, all datasets were of sufficient quality for further processing. Figure 2 provides an overview of the images on the 1st time point for all 15 subjects. Figure 3 shows images from two time points for the dataset with the lowest SNR and a representative dataset with high SNR. For both the lower and higher SNR datasets, the anatomical registration was good.

### DTI parameters

**Fig. 4:**
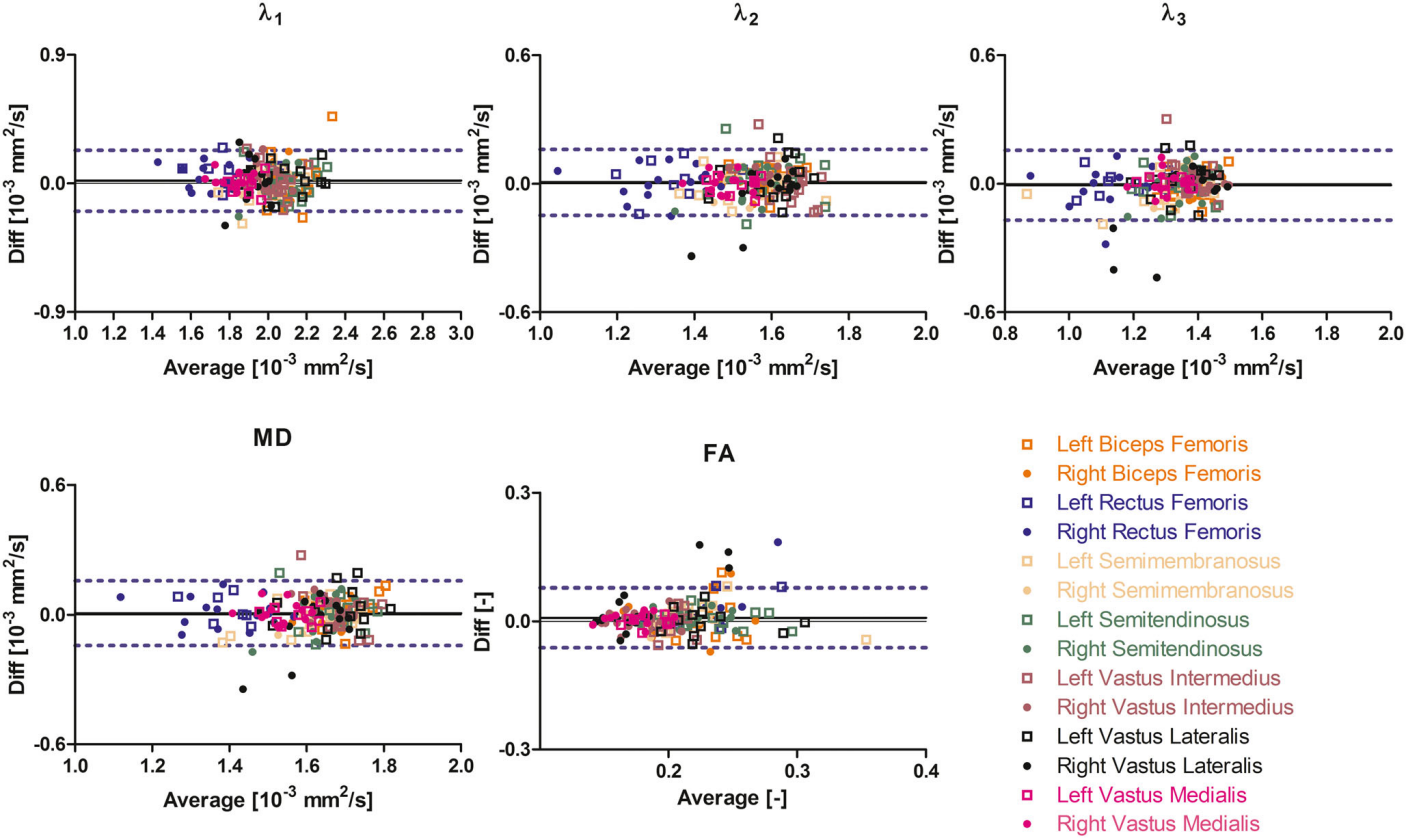
Bland-Altman plots of the IVIM-corrected DTI parameters per muscle. The 95% confidence interval and the mean of the paired difference are indicated by dashed and solid lines

**Fig. 5:**
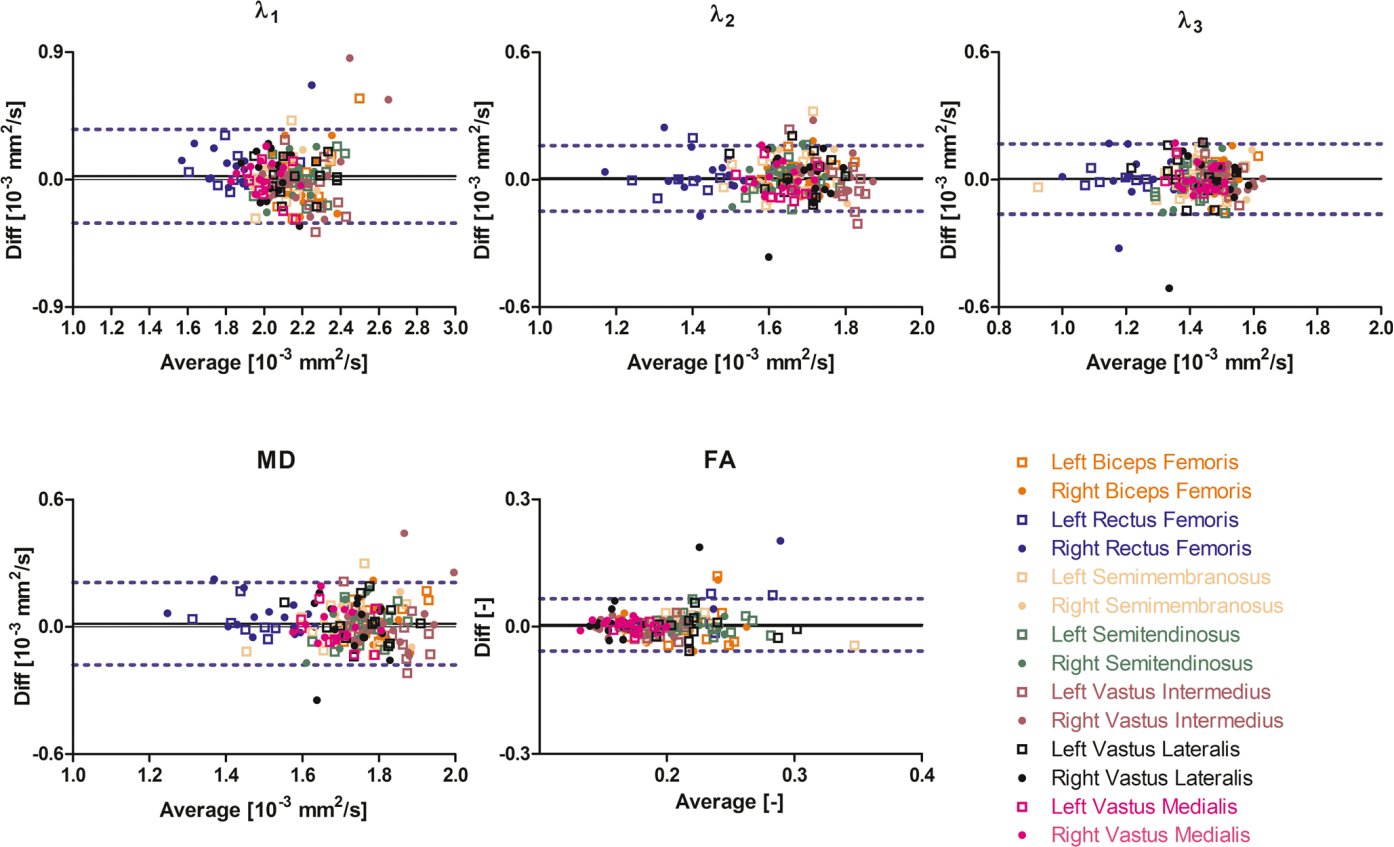
Bland-Altman plots of the standard DTI parameters per muscle. The 95% confidence interval and the mean of the paired difference are indicated by dashed and solid lines

Average values and standard deviation of the individual DTI parameters per muscle are reported in Table 2. The mean values for all IVIM-corrected DTI parameters except FA were lower than the mean values of the standard DTI parameters due to the absence of perfusion contamination. The Bland-Altman analysis showed good agreement between time points 1 and 2 for all DTI parameters, except FA for both standard and IVIM-corrected data (Figs. 4 and 5). wsCV values varied between 2.2 and 37.7% for IVIM-corrected DTI data and between 2.9 and 32.3% for the standard DTI data (Table 3).

**Table 2:** Mean DTI indices (10^−3^ mm^2^/s) for all subjects at time point 1

Standard						IVIM				
Muscle	*λ*_1_ (10^−3^ mm^2^/s)	*λ*_2_ (10^−3^ mm^2^/s)	*λ*_3_ (10^−3^ mm^2^/s)	MD (10^−3^ mm^2^/s)	FA (−)	*λ*_1_ (10^−3^ mm^2^/s)	*λ*_2_ (10^−3^ mm^2^/s)	*λ*_3_ (10^−3^ mm^2^/s)	MD (10^−3^ mm^2^/s)	FA (−)
BFL	2.23 ± 0.20	1.70 ± 0.07	1.47 ± 0.08	1.80 ± 0.1	0.21 ± 0.04	2.14 ± 0.16	1.61 ± 0.06	1.38 ± 0.08	1.71 ± 0.08	0.22 ± 0.04
BFR	2.19 ± 0.14	1.67 ± 0.07	1.48 ± 0.06	1.78 ± 0.07	0.20 ± 0.04	2.06 ± 0.10	1.57 ± 0.06	1.39 ± 0.06	1.67 ± 0.05	0.21 ± 0.04
RFL	1.88 ± 0.19	1.41 ± 0.13	1.21 ± 0.11	1.50 ± 0.13	0.23 ± 0.05	1.82 ± 0.15	1.36 ± 0.12	1.15 ± 0.11	1.44 ± 0.11	0.24 ± 0.05
RFR	1.89 ± 0.21	1.43 ± 0.1	1.24 ± 0.12	1.52 ± 0.10	0.21 ± 0.05	1.74 ± 0.14	1.32 ± 0.12	1.14 ± 0.14	1.40 ± 0.12	0.22 ± 0.05
SML	2.10 ± 0.16	1.65 ± 0.11	1.37 ± 0.15	1.71 ± 0.13	0.22 ± 0.04	1.98 ± 0.14	1.55 ± 0.11	1.28 ± 0.16	1.60 ± 0.13	0.23 ± 0.04
SMR	2.08 ± 0.12	1.68 ± 0.1	1.48 ± 0.08	1.75 ± 0.10	0.18 ± 0.01	1.97 ± 0.09	1.56 ± 0.07	1.38 ± 0.08	1.64 ± 0.08	0.19 ± 0.02
STL	2.22 ± 0.15	1.66 ± 0.09	1.41 ± 0.08	1.76 ± 0.08	0.23 ± 0.03	2.14 ± 0.11	1.60 ± 0.1	1.34 ± 0.08	1.69 ± 0.07	0.24 ± 0.03
STR	2.16 ± 0.12	1.64 ± 0.09	1.44 ± 0.1	1.75 ± 0.09	0.22 ± 0.03	2.05 ± 0.12	1.53 ± 0.09	1.34 ± 0.10	1.64 ± 0.1	0.23 ± 0.02
VIL	2.19 ± 0.11	1.76 ± 0.06	1.49 ± 0.04	1.81 ± 0.06	0.19 ± 0.02	2.05 ± 0.08	1.65 ± 0.06	1.39 ± 0.05	1.70 ± 0.05	0.20 ± 0.02
VIR	2.25 ± 0.30	1.76 ± 0.07	1.54 ± 0.04	1.85 ± 0.13	0.16 ± 0.02	1.99 ± 0.08	1.61 ± 0.05	1.41 ± 0.06	1.67 ± 0.05	0.17 ± 0.03
VLL	2.20 ± 0.16	1.69 ± 0.09	1.42 ± 0.09	1.77 ± 0.09	0.22 ± 0.04	2.11 ± 0.16	1.61 ± 0.1	1.35 ± 0.09	1.69 ± 0.11	0.23 ± 0.04
VLR	2.05 ± 0.09	1.69 ± 0.09	1.47 ± 0.12	1.74 ± 0.09	0.17 ± 0.04	1.92 ± 0.10	1.57 ± 0.13	1.33 ± 0.18	1.61 ± 0.13	0.20 ± 0.06
VML	2.00 ± 0.09	1.62 ± 0.07	1.42 ± 0.06	1.68 ± 0.07	0.18 ± 0.01	1.88 ± 0.08	1.51 ± 0.06	1.31 ± 0.06	1.57 ± 0.06	0.18 ± 0.02
VMR	2.02 ± 0.11	1.62 ± 0.06	1.43 ± 0.06	1.69 ± 0.07	0.17 ± 0.02	1.83 ± 0.09	1.48 ± 0.05	1.30 ± 0.06	1.54 ± 0.06	0.18 ± 0.02

DTI indices are given for standard and IVIM modeling of the diffusion attenuation as function of *b* value. *RFR*, right rectus femoris; *RFL*, left rectus femoris; *VMR*, right vastus medialis; *VML*, left vastus medialis; *VIR*, right vastus intermedius; *VIL*, left vastus intermedius; *VLR*, right vastus lateralis; *VLL*, left vastus lateralis; *STR*, right semitendinosus; *STL*, left semitendinosus; *BFR*, right biceps femoris long head; *BFL*, left biceps femoris long head; *SMR*, right semimembranosus; *SML*, left semimembranosus

**Table 3:** wsCV (%) per muscle for each DTI parameter determined by standard and IVIM modeling

Standard						IVIM				
Muscle	*λ*_1_*	*λ*_2_	*λ*_3_	MD*	FA	*λ*_1_*	*λ*_2_	*λ*_3_	MD*	FA
BFL	9.2	3.4	4.4	4.8	18.7	8.0	3.4	4.9	4.2	19.7
BFR	8.3	4.5	5.2	5.4	18.5	5.6	3.1	4.1	3.0	19.2
RFL	7.3	6.1	2.9	4.7	17.3	5.9	6.7	5.3	4.5	17.4
RFR	9.9	6.6	9.1	5.2	25.9	5.1	5.7	8.1	5.0	22.3
SML	7.9	6.7	5.6	6.5	10.2	5.5	4.7	5.4	4.5	14.1
SMR	4.1	5.6	5.0	4.5	8.7	3.4	4.0	4.9	3.5	9.9
STL	5.7	4.6	5.0	4.3	10.2	5.1	6.5	4.7	4.8	9.3
STR	6.2	5.1	6.7	5.8	6.8	5.5	5.1	7.0	5.5	8.7
VIL	7.7	6.1	4.8	5.9	9.8	5.8	6.6	7.0	5.9	13.6
VIR	13.5	5.2	4.1	8.1	8.9	4.7	2.5	2.3	2.9	12
VLL	5.8	5.9	6.9	5.6	12.6	5.5	6.3	7.1	5.6	13.2
VLR	8.0	7.5	10.2	7.7	32.3	7.7	7.9	11.8	7.8	37.7
VML	7.2	4.8	4.3	5.4	6.8	3.6	2.8	2.2	2.6	6.9
VMR	4.7	4.3	4.9	4.5	7.3	2.7	3.1	4.4	3.1	8.1

*RFR*, right rectus femoris; *RFL*, left rectus femoris; *VMR*, right vastus medialis; *VML*, left vastus medialis; *VIR*, right vastus intermedius; *VIL*, left vastus intermedius; *VLR*, right vastus lateralis; *VLL*, left vastus lateralis; *STR*, right semitendinosus; *STL*, left semitendinosus; *BFR*, right biceps femoris long head; *BFL*, left biceps femoris long head; *SMR*, and right semimembranosus; *SML*, left semimembranosus. *Significant difference *p* < 0.05

The MD had the highest repeatability (4.5% IVIM; 5.6% standard), followed by *λ*_2_ (4.9% IVIM; 5.5% standard), *λ*_1_ (5.3% IVIM; 7.5% standard), and *λ*_3_ (5.7% IVIM; 5.7% standard). FA had the lowest repeatability of all DTI parameters, 15.2% for the IVIM-corrected data and 13.9% for the standard analysis. For an overview of the wsCV and its standard deviation (SD) per DTI parameter, calculated as the mean of all muscles per DTI parameter, see Table 4. The mean wsCV values over all muscles of *λ*_1_ (*p* = 0.003) and MD (*p* = 0.02) were significantly lower for the IVIM-corrected data. The wsCV values of *λ*_2_ (*p* = 0.14), *λ*_3_ (*p* = 0.96), and FA (*p* = 0.05) for the IVIM-corrected data were also lower but not significantly. The mean wsCV (IVIM-corrected data) was comparable for the left and right legs (Table 3).

**Table 4:** Mean wsCV and standard deviation (%) per DTI parameter

DTI parameter	IVIM	Standard
MD	4.5 ± 1.4*	5.6 ± 1.2*
FA	15.2 ± 8.0	13.9 ± 7.8
*λ*_1_	5.3 ± 1.5*	7.5 ± 2.4*
*λ*_2_	4.9 ± 1.8	5.5 ± 1.1
*λ*_3_	5.7 ± 2.5	5.7 ± 2.0

*Significant difference *p* < 0.05

The IVIM-corrected DTI data showed slightly lower values for the minimal detectable difference compared with the standard analysis. This was the case for all DTI parameters except FA (Supplemental Table 5).

### Additional B1^+^ calibration

For this group, 2 muscles out of 140 muscles (5 subjects × 7 muscles × 2 legs × 2 time points) were excluded from the analysis due to insufficient SNR (< 20). Mean SNR (all muscles) was 50.0 ± 12.2 for the 5 subjects with additional calibration vs. 37.6 ± 16.9 for the 10 standard subjects. The mean wsCV values per DTI parameter for the IVIM-corrected data were lower for these 5 subjects compared with those for the 10 subjects with the regular protocol (Supplemental Table 3). This was significant for *λ*_2_ (*p* = 0.04), *λ*_3_ (*p* = 0.03), and MD (*p* = 0.04), but not for *λ*_1_ (*p* = 0.06) and FA (*p* = 0.1).

### SNR in relation to DTI indices

**Fig. 6:**
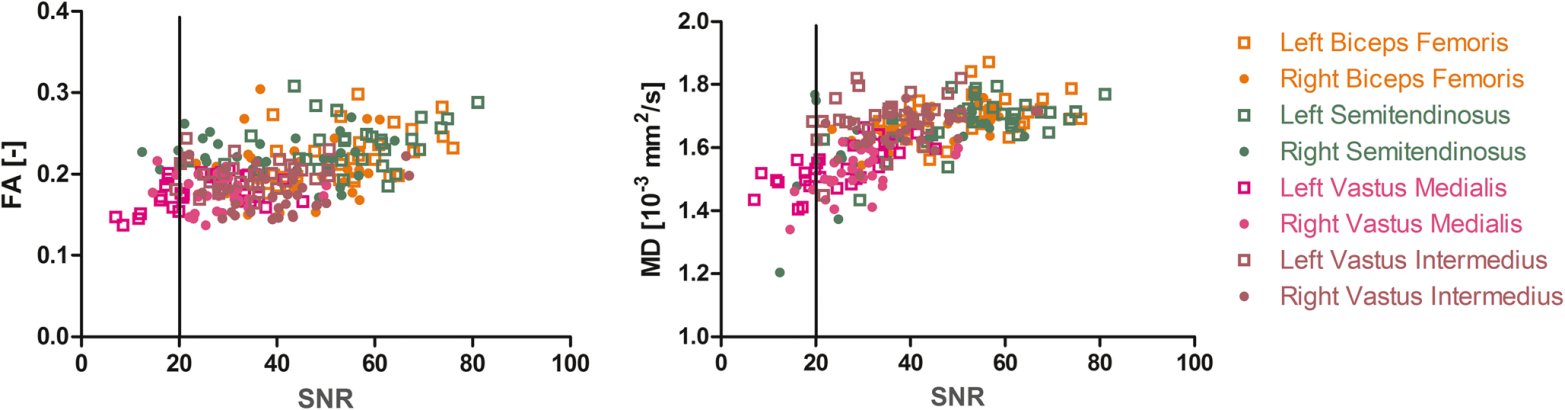
IVIM-corrected FA (top graph) and MD (bottom graph) as function of SNR for each individual muscle

The IVIM-corrected MD and FA were plotted against the SNR for 2 hamstring (BF and ST) and 2 quadriceps (VM and VI) muscles of the left and right legs (Fig. 6) in order to detect potential SNR bias. An underestimation of MD was present in the lower SNR range (SNR < 20) but no overestimation of FA.

## Discussion

Our research showed good repeatability of the DTI parameters in the upper leg muscles using a clinically applicable protocol suited for imaging of both legs simultaneously. The repeatability became even slightly better after IVIM correction for perfusion. Furthermore, performing an additional B1^+^ calibration improved SNR and repeatability of the DTI parameter estimations. Lastly, we found that the data quality (SNR) and repeatability (wsCV) of the DTI parameters were similar for the left and right legs.

### Data quality

The overall quality of the images was visually judged good. However, chemical shift artifacts by residual unsuppressed fat and shading due to B1^+^ field inhomogeneities were regionally observed. The direction of the fat shift was chosen from anterior to posterior ensuring that residual fat signal would not shift into the commonly injured hamstring muscles. However, this resulted in some fat artifacts in the quadriceps muscles. The residual fat signal is caused by suboptimal fat suppression due to B0-field inhomogeneities. A research pre-pulse (saturation pulse) did improve suppression of the lipid olefinic resonance, but at the cost of decreased overall data quality caused by direct saturation of water, as this lipid resonance is very close to the water peak.

B1^+^ inhomogeneities due to constructive and destructive interference of the transmit field are a common problem at ultrahigh fields (≥ 7 T). However, B1^+^ inhomogeneities can also lead to regional loss of signal (shading artifacts) at 3 T in particular in the thighs, hips, and pelvis region. In this work, we employed dielectric pads to locally augment the transmit field and reduce the shading artifacts. Although in many cases the shading was still slightly visible, the pads were capable of recovering most of the signal as evidenced from similar high SNR levels for both the right (41.8 ± 16.0) and left (41.7 ± 17.1) legs.

To investigate whether we could further optimize the transmit field homogeneity, we performed additional manual B1^+^ calibration prior to the DTI sequence for five subjects. This further improved signal homogeneity, reduced the fat and chemical shift artifacts, and increased SNR with fewer muscles excluded, i.e., 2 out of 140 muscles versus 48 out of 280 muscles. Importantly, the wsCV values per DTI parameter were lower, suggesting higher repeatability. This is also in line with the simulation work of Damon [16]. Additionally, Filli et al performed twofold and threefold acceleration of the DTI sequence using simultaneous multi-slice acquisition, with the twofold acceleration achieving similar fibertracking and quantitative results as the conventional DTI sequence [27]. This could decrease the scanning time and further increase the clinical applicability of this protocol.

### DTI parameters

The muscle DTI parameter values were comparable with those reported in previous studies [28, 29]. IVIM correction reduced all parameter values except FA. This is in line with previous work in the human calf, which showed that not accounting for perfusion leads to MD and eigenvalue overestimation and FA underestimation [20].

We found that the wsCV values for the three eigenvalues and MD were on average lower after IVIM modeling. In contrast, wsCV values for FA were on average higher after IVIM correction. A similar pattern was observed when comparing our IVIM-corrected diffusion parameters to previous work in forearm muscles [28]. Furthermore, the minimal detectable difference values for both the eigenvalues and MD were lower for IVIM-corrected DTI data compared with those for standard analysis. Taken these findings combined suggests that IVIM modeling positively affects repeatability of the diffusion parameters with exception of FA and as a result improves the detection limit of the technique. Previous research has shown that changes in DTI parameters after exercise and/or injury are mostly present in MD, *λ*_2_, and *λ*_3_ (radial diffusivity) [11, 30, 31]. Therefore, achieving high repeatability for MD, RD, and the eigenvalues is essential. Hence, we recommend the use of IVIM correction for its positive effect on the repeatability of MD and the eigenvalues. Importantly, DTI parameters for the left and right legs had similar repeatability, indicating that bilateral imaging protocol used here can accurately quantify diffusion parameters in both legs simultaneously and the contralateral leg thus can be used as internal control.

### Study limitations

Some limitations of the study should be acknowledged. Firstly, we included 13 males and only 2 females. This was done to reflect the demographics of our hamstring injury patients, which are predominantly male soccer players. Men have a higher incidence rate of hamstring injuries compared with women, even when practicing the same sport [32]. Secondly, our subjects were not rested for 30 min prior to scanning as was previously recommended [33], which may have increased the variance. Furthermore, we did not collect data on the time interval between the last exercise session of the subjects and the MRI examinations. Exercise and post-exercise muscle edema might have caused further variance in DTI parameters [30].

Additionally, SNR values were estimated from the PCA denoising and not from a separate noise map acquisition. This may have resulted in a systemic underestimation of the SNR and exclusion of some datasets based on the strict SNR threshold of 20. Better SNR estimates would improve the repeatability of the DTI parameters due to the known correlation between SNR and repeatability [16]. Furthermore, an additional B1^+^ calibration was only performed for 5 subjects. We believe that repeatability would have been even higher if this additional B1^+^ calibration had been performed for all subjects.

## Conclusion

DTI parameters (MD and eigenvalues), except FA, were repeatable. IVIM correction and additional B1^+^ calibration further improved the repeatability. On average, wsCV values were slightly lower than the values reported in previous studies. We plan to use this protocol in future studies on subjects with leg muscle injury.

## Electronic supplementary material

ESM 1 (DOCX 3675 kb)

## Abbreviations

BFL: Left biceps femoris long head
BFR: Right biceps femoris long head
IVIM: Intravoxel incoherent motion
MD: Mean diffusivity
RFL: Left rectus femoris
RFR: Right rectus femoris
ROI: Region of interest
SML: Left semimembranosus
SMR: Right semimembranosus
SNR: Signal-to-noise ratio
STL: Left semitendinosus
STR: Right semitendinosus
VIL: Left vastus intermedius
VIR: Right vastus intermedius
VLL: Left vastus lateralis
VLR: Right vastus lateralis
VML: Left vastus medialis
VMR: Right vastus medialis
*λ*_1_: First Eigenvalue
*λ*_2_: Second eigenvalue
*λ*_3_: Third eigenvalue
