# Supplementary material for: The repeatability of bilateral diffusion tensor imaging (DTI) in the upper leg muscles of healthy adults
Source: Eur Radiol. 2019 Nov 8;30(3):1709–18. doi: 10.1007/s00330-019-06403-5 (PMC7033061; doi:10.1007/s00330-019-06403-5)
Supplement: Supplementary file 1 — (DOCX 3675 kb) [file 330_2019_6403_MOESM1_ESM.docx]

**Supplementary material**

**Supplemental Table 1:** Number of muscles excluded per subject.

| **Subject** | **# excluded muscles** |
| --- | --- |
| **1** | 8 |
| **2** | 6 |
| **3** | 0 |
| **4** | 4 |
| **5** | 2 |
| **6** | 10 |
| **7** | 8 |
| **8** | 6 |
| **9** | 2 |
| **10** | 2 |
| **11** | 2 |
| **12** | 0 |
| **13** | 0 |
| **14** | 0 |
| **15** | 0 |

**Supplemental Table 2:** Included and excluded muscles with corresponding SNR.

| **Muscle** | **#included** | **SNR_included_** | **#excluded** | **SNR_excluded_** |
| --- | --- | --- | --- | --- |
| **BFL** | 30 | 53.9 ± 11.9 | 0 | - |
| **BFR** | 30 | 44.1 ± 10.8 | 0 | - |
| **RFL** | 16 | 53.8 ± 16.6 | 7 (14) | 14 ± 5.3 |
| **RFR** | 30 | 58.1 ± 15.4 | 0 | - |
| **SML** | 30 | 47.6 ± 14.4 | 0 | - |
| **SMR** | 20 | 42.7 ± 15.0 | 5 (10) | 14.5 ± 5 |
| **STL** | 30 | 55.1 ± 13.6 | 0 | - |
| **STR** | 26 | 41.3 ± 13.2 | 2 (4) | 17.9 ± 2.7 |
| **VIL** | 28 | 35.8 ± 8.9 | 1 (2) | 19.34 |
| **VIR** | 30 | 39.5 ± 10.3 | 0 | - |
| **VLL** | 26 | 40.1 ± 10.8 | 2 (4) | 17.5 ± 0.7 |
| **VLR** | 30 | 49.2 ± 13.9 | 0 | - |
| **VML** | 18 | 31.4 ± 6.5 | 6 (12) | 16.4 ± 4.1 |
| **VMR** | 26 | 29.9 ± 6.9 | 2 (4) | 17.5 ± 2.8  99 |
| **Total** | 370 | 44.5 ± 8.9 | 25 (50) |  |

Number of included and excluded muscles per muscle, and mean SNR of included and excluded muscles per muscle. In case of insufficient SNR at one time point, the muscle was automatically excluded for the other time point (regardless of SNR). The SNR noted below for the excluded muscles is based on the first exclusion. The total amount of excluded muscles is shown in brackets.

Right Rectus Femoris (RFR), Left Rectus Femoris (RFL), Right Vastus Medialis (VMR), Left Vastus Medialis (VML), Right Vastus Intermedius (VIR), Left Vastus Intermedius (VIL), Right Vastus Lateralis (VLR), ), Left Vastus Lateralis (VLL), Right Semitendinosus (STR), Left Semitendinosus (STL), Right Biceps Femoris Long head (BFR), Left Biceps Femoris Long head (BFL) and Right Semimembranosus (SMR), Left Semimembranosus (SML).

**Supplemental Table 3:** Lower wsCV values in subjects with additional B1^+^ calibration.

| **DTI parameter** | **IVIM 5 subjects** | **IVIM 10 subjects** |
| --- | --- | --- |
| **MD** | 3.4 ± 1.5* | 4.8 ± 1.8* |
| **FA** | 9.5 ± 5.5 | 15.3 ± 10 |
| **λ_1_** | 4.2 ± 1.8 | 5.7 ± 1.8 |
| **λ_2_** | 3.5 ± 1.5* | 5 ± 2.2* |
| **λ_3_** | 3.8 ± 2.0* | 6. 1 ± 3.1* |

wsCV values and standard deviation (%) per DTI parameter. The wsCV values are based on the IVIM corrected data of the 5 subjects with one additional manual B1^+^ calibration and the 10 subjects with the regular protocol. *Significant difference p<0.05

**Supplemental Table 4:** wsCV values (%) per muscle per IVIM corrected DTI parameter for 5 subjects with an additional B1^+^ calibration scan.

| **IVIM** | **5 subjects** | | | |  |  |
| --- | --- | --- | --- | --- | --- | --- |
| **Muscle** | **λ_1_** | **λ_2_** | **λ_3_** | **MD** | | **FA** |
| **BFL** | 5.4 | 0.9 | 4.8 | 4.4 | | 13.4 |
| **BFR** | 4.7 | 2.1 | 3.8 | 2.9 | | 15.6 |
| **RFL** | 7.3 | 6.2 | 6.3 | 5.4 | | 21.1 |
| **RFR** | 5.1 | 5.5 | 5.2 | 4.9 | | 2.3 |
| **SML** | 4.8 | 3.7 | 2.6 | 3.3 | | 13.2 |
| **SMR** | 3.3 | 2.2 | 4.4 | 2.5 | | 11.6 |
| **STL** | 4.7 | 5.3 | 2.2 | 3.9 | | 8.6 |
| **STR** | 2.6 | 4.7 | 4.4 | 2.6 | | 8.7 |
| **VIL** | 3.5 | 2.3 | 3.5 | 3.6 | | 6.4 |
| **VIR** | 2.9 | 2.5 | 1.3 | 2.2 | | 3.6 |
| **VLL** | 7.8 | 4.7 | 8.3 | 6.8 | | 13.3 |
| **VLR** | 2.4 | 3 | 2.0 | 2.2 | | 3.1 |
| **VML** | 3.2 | 3.3 | 1.9 | 1.8 | | 8.6 |
| **VMR** | 1.7 | 3.1 | 2.3 | 1.7 | | 3.3 |

Right Rectus Femoris (RFR), Left Rectus Femoris (RFL), Right Vastus Medialis (VMR), Left Vastus Medialis (VML), Right Vastus Intermedius (VIR), Left Vastus Intermedius (VIL), Right Vastus Lateralis (VLR), ), Left Vastus Lateralis (VLL), Right Semitendinosus (STR), Left Semitendinosus (STL), Right Biceps Femoris Long head (BFR), Left Biceps Femoris Long head (BFL) and Right Semimembranosus (SMR), Left Semimembranosus (SML).

**Supplemental Table 5**: Slightly Lower minimal detectable difference per muscle for

IVIM data.

|  | |  | **Standard** |  | |  | |  | **IVIM** | |  |
| --- | --- | --- | --- | --- | --- | --- | --- | --- | --- | --- | --- |
| **muscle** | **λ_1_**  [10^-3^ mm^2^/s] | **λ_2_**  [10^-3^ mm^2^/s] | **λ_3_**  [10^-3^ mm^2^/s] | **MD**  [10^-3^ mm^2^/s] | **FA**  [-] | | **λ_1_**  [10^-3^ mm^2^/s] | **λ_2_**  [10^-3^ mm^2^/s] | **λ_3_**  [10^-3^ mm^2^/s] | **MD**  [10^-3^ mm^2^/s] | **FA**  [-] |
| **BFL** | 0.40 | 0.11 | 0.13 | 0.17 | 0.08 | | 0.33 | 0.11 | 0.13 | 0.14 | 0.09 |
| **BFR** | 0.36 | 0.15 | 0.15 | 0.19 | 0.07 | | 0.23 | 0.10 | 0.11 | 0.10 | 0.08 |
| **RFL** | 0.26 | 0.17 | 0.07 | 0.14 | 0.07 | | 0.21 | 0.18 | 0.12 | 0.13 | 0.08 |
| **RFR** | 0.36 | 0.18 | 0.22 | 0.15 | 0.10 | | 0.17 | 0.15 | 0.18 | 0.14 | 0.09 |
| **SML** | 0.32 | 0.21 | 0.15 | 0.22 | 0.04 | | 0.21 | 0.14 | 0.14 | 0.14 | 0.06 |
| **SMR** | 0.16 | 0.18 | 0.14 | 0.15 | 0.03 | | 0.13 | 0.12 | 0.13 | 0.11 | 0.04 |
| **STL** | 0.25 | 0.15 | 0.14 | 0.15 | 0.05 | | 0.21 | 0.20 | 0.12 | 0.16 | 0.04 |
| **STR** | 0.26 | 0.16 | 0.19 | 0.20 | 0.03 | | 0.22 | 0.15 | 0.19 | 0.18 | 0.04 |
| **VIL** | 0.33 | 0.21 | 0.14 | 0.21 | 0.04 | | 0.23 | 0.21 | 0.19 | 0.20 | 0.05 |
| **VIR** | 0.58 | 0.18 | 0.12 | 0.29 | 0.03 | | 0.18 | 0.08 | 0.06 | 0.09 | 0.04 |
| **VLL** | 0.25 | 0.19 | 0.19 | 0.19 | 0.06 | | 0.22 | 0.2 | 0.19 | 0.18 | 0.06 |
| **VLR** | 0.32 | 0.25 | 0.29 | 0.26 | 0.10 | | 0.29 | 0.24 | 0.31 | 0.25 | 0.13 |
| **VML** | 0.28 | 0.15 | 0.12 | 0.18 | 0.02 | | 0.13 | 0.08 | 0.06 | 0.08 | 0.03 |
| **VMR** | 0.18 | 0.14 | 0.14 | 0.15 | 0.02 | | 0.10 | 0.09 | 0.11 | 0.09 | 0.03 |

Right Rectus Femoris (RFR), Left Rectus Femoris (RFL), Right Vastus Medialis (VMR), Left Vastus Medialis (VML), Right Vastus Intermedius (VIR), Left Vastus Intermedius (VIL), Right Vastus Lateralis (VLR), ), Left Vastus Lateralis (VLL), Right Semitendinosus (STR), Left Semitendinosus (STL), Right Biceps Femoris Long head (BFR), Left Biceps Femoris Long head (BFL) and Right Semimembranosus (SM), Left Semimembranosus (SML).

**
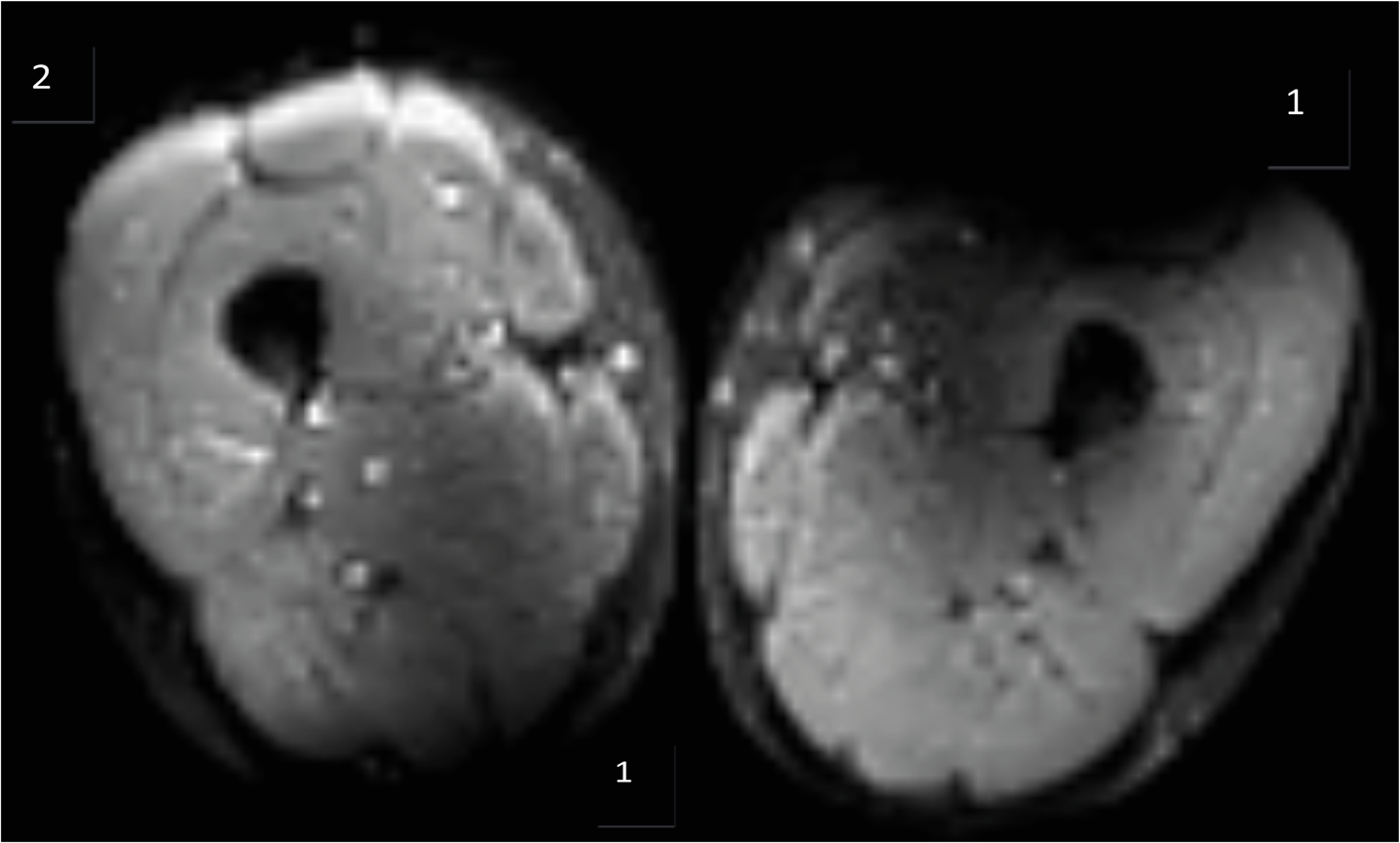
Supplemental Figure 1:** Axial DTI (b-value = 0 s/mm^2^) of subject 6 indicating (1) shading artifacts due to B1^+^ inhomogeneities in the upper quadrant of the left leg and lower quadrant of the right leg, and (2) a chemical shift artefact due to unsuppressed fat in the upper quadrant of the right leg.
